# Natural hazard insurance demand: A systematic review

**DOI:** 10.4102/jamba.v14i1.1223

**Published:** 2022-05-27

**Authors:** Farai B. Mushonga, Syden Mishi

**Affiliations:** 1 Department of Economics, Faculty of Business and Economic Sciences, Nelson Mandela University, Gqeberha, South Africa

**Keywords:** natural hazard insurance demand, psychological effect, willingness to pay, risk preference, risk perception

## Abstract

The mitigation of natural hazard costs such as loss of property, life, crops and medical costs can be achieved through the adoption of insurance. It is, however, not clear whether there is corresponding demand for insurance given the increasing frequency and veracity of natural hazards, especially in South Africa. This study follows the guideline of Preferred Reporting items for Systematic Review and Meta-analysis Protocols (PRISMA-P) to identify the relevant works on the subject. A total of 645 articles emerged on initial search and after screening, 39 remained which have been reviewed in this study. Reviewing the studies and conflating with the study objectives, the following themes emerged for discussion on demand for natural hazard insurance, is there demand for natural hazard insurance?; psychology of decision-making; risk perception; risk preference and willingness to pay. The study found that studies of demand for insurance have identified that there is low demand for tailor-made insurance products for natural hazards. Further analysis of the demand revealed that normative and descriptive decision-making of buying natural hazard insurance is part of the psychological factors that determine demand. Whilst risk preference and perception have sub-attributes that affect their impact on demand such as experience, age and salience to natural hazards in communities. Whilst willingness to pay is also a broad concept which is analysed using both monetary and non-monetary factors in literature, the results also identified that there is a huge gap in literature in terms of studies that cover risk preference and perception in Africa and in the Southern African Development Community (SADC) region.

## Introduction

There has been a significant increase in natural hazards and disasters over the recent past, all attributable to climate change and the significant rise in population (Schwarze et al. 2011). Although many hazards do not end up disastrously, the significant number of disasters is worrisome. The hazards have been increasing in frequency and level of risk, which may enable modelling compensation of insurance type (Schwarze et al. 2011). Although in the context of disaster management, effort is to reduce the risk of a hazard turning into a disaster, often, the need for coping strategies based on one of the three components of risk, cost (other two are threat and vulnerability) is needed. As more and more individuals and property are vulnerable to hazards, in their increasing threat, the cost element rises, especially when it is borne by governments as is the case in many regions (Schwarze et al. 2011). The market mechanisms can be used to help cover the costs; however, the appetite for such products is not well-known (Schwarze et al. 2011).

Disasters are a serious disruption of the functioning of a community or a society at any scale due to hazardous events interacting with conditions of exposure, vulnerability and capacity, leading to human, material, economic and environmental loses and impacts. These disasters can be classified into two which are a slow-onset disaster and a sudden-onset disaster. Kellenberg and Mobarak (2011) state that a slow-onset disaster can be defined as a disaster that emerges gradually overtime such as drought, desertification, sea-level rise, epidemic disease. Whilst a sudden onset disaster is one that is caused by hazardous events that emerges quickly or unexpectedly such as earthquakes, volcanic eruptions, flash floods, chemical explosions and critical infrastructural failures (Kellenberg & Mobarak 2011). Disasters can be further classified as natural disasters and other disasters. Natural disasters are the ones that originate in the physical environment, and they can be further sub-divided into geological (earthquakes, tsunamis, volcanoes, dry mass movement), biological (epidemics, insects, infestations) climatological (drought, extreme temperatures, wildfires), hydrological (flood wet mass movement), meteorological disasters (storms) (Kellenberg & Mobarak 2011). The causes of these natural disasters include climate change, anthropogenic force and the natural phenomena (act of God).

According to Wouter Botzen, Deschenesa and Sanders (2020), the natural hazards in 2017 were the costliest on recorded natural hazards in the world with a staggering cost of $3 trillion. It is interesting to note that out of that total world cost, the United States dollars (USD) 134–13824 (billion) was the only insured amount in the year 2017 (Forsyth, Walls & Fortune 2019). Natural hazards have affected the growth and development of the world economy in which Baarsch et al. (2020) found that it accounts for 10% – 15% of its gross domestic product (GDP) per capita loss. The increase in global temperature has increased the frequency of the previously low frequency high impact natural hazards, and high frequency medium impact natural hazards. Studies that have explored the impact of these natural events on GDP include study by Botzen et al. (2020) who identified that in the short run, there is a negative relationship with economic activity.

Whilst Gignoux (2016) states that the multiplier of investments to recoup the lost properties and infrastructure leads to future economic growth and increase in economic activity. Snippet from a Southern African Perspective include Mozambique, Zimbabwe, Malawi, Madagascar, and other Southern African countries commonly affected by a set of natural hazards such as droughts, fire, extreme temperatures and storms below. According to United Nations (UN) report, in 2019, approximately 23 million, 730 000 and 165 million people in Zimbabwe, Malawi and Mozambique, respectively, were affected by the combined effects of droughts, cyclones and have acute food insecurity. According to the UN report on natural hazards and cyclones, the impact cost of flooding was approximately USD 2.2 billion in 2019 in Southern Africa.

The growing social and economic costs of natural hazards are a growing threat to the achievement of sustainable development, economic growth and efforts of alleviation of poverty as noted in the study by (Fernandez & Ceacero-Moreno 2021). Amongst the post natural hazard experiences, there is an increase in stressors and gender-based violence of girls and women (Thurston, Stöckl & Ranganathan 2021). Climate shocks erode assets and adaptive ways of people in societies leading to migration and increase in protests in the receiving districts (Petrova 2021). Insurance against the impact of natural hazards is one of the ways of strengthening the adaptive positions of communities and reduce the impact of these events (Petrova 2021). However, it is interesting to note that there are countries that either have a low penetration of natural hazards’ insurance or have no such insurance. For instance, according to Lester (2014), South Africa is by far the largest African insurance market generating about USD44b in premiums, however, it is interesting to note that there has been few to no existence of natural hazard insurance with low penetration of natural hazards insurance products in South Africa.

Reynaud and Nguyen (2018) explain that the slow penetration of insurance demand is attributed to government aid, lack of awareness and adverse selection (Reynaud & Nguyen 2018). Roder, Hudson and Tarolli (2019) have noted that the mare existence of these natural hazard insurance has been affected by moral hazard. It is difficult to identify the homogeneous and heterogeneity of determinants of demand for insurance of natural hazards because the studies are classified based on the type of natural hazards from earthquake, flooding, drought and wildfires and therefore a systematic literature review will help to have a comprehensive understanding. Many studies in literature have attributed slow penetration to unwillingness to pay for flood insurance, which was analysed in Vietnam, by Navrud, Tuan and Tinh (2012) and found that households are willing to contribute to flood prevention programmes at an average of 6.7 person-days per year and per household, whilst other studies attribute demand to risk preference and perception. Other studies have identified that when dealing with insurance demand, it is important to analyse the psychology of decision-making and a systematic literature review will provide an empirical in-depth understanding of these in relation to natural hazard insurance demand.

The studies of the demand side of natural hazard insurance have been widely undertaken using two main principles, which are the choice experiments and the contingent valuation approach. The contingent valuation technique is limited in terms of conditions that can be presented, whilst the choice experiments can be extensive. Although existing literature shows a plethora of studies on the demand for insurance of a range of factors of natural hazards and several domains, no systematic review exists on the subject in relation to analysing the factors that derive demand of hazard insurance. Relevant questions are: (1) what are the psychological factors that affect natural hazard insurance? (2) How does risk preference affect insurance demand; (3) how does risk perception affect insurance demand? and (4) what are the determinants of willingness to pay for natural hazard insurance? Therefore, as the first step, we conducted a systematic literature review of all the studies explaining the psychology of uncertainty and decision-making biases for busying insurance, risk preference, risk perception and willingness to pay. The aim of this study is to have a comprehensive understanding of both the monetary and non-monetary factors of natural hazard insurance.

## Review principles, materials and methods

A systematic review was conducted on the demand for natural hazard insurance. The review focuses on the natural hazard such as earthquakes, cyclones, floods and droughts. To ensure reproducibility and transparency of our findings, this review follows the Preferred Reporting Items for Systematic Review and Meta-Analysis Protocols (PRISMA-P) guidelines which is a scientific procedure that has been followed in the study by (Habiba, Shaw & Takeuchi 2012; Hezam & Nayeem 2021; Kumar et al. 2021; Thurston et al. 2021) unlike Matunhu, Mago and Matunhu (2022). The systematic approach has been applied to related studies and improves scientificity of the review (see, e.g., Djalante 2018; Estevão & Costa 2020; Islam et al. 2020; Kalanlar 2021; Sohrabizadeh et al. 2021; Suk et al. 2020).

To do this, a comprehensive literature search was conducted on the following electronic databases: Google Scholar, ScienceDirect, Taylor and Francis, Emerald, Wiley, Springer and Journal storage. Search parameters include the following descriptions: ‘insurance’, ‘insurance demand’, ‘demand for natural hazard insurance’, ‘demand for flood, earthquake, cyclone and drought insurance’, ‘natural hazard costs mitigation’, ‘decision-making in the face of uncertainty’, ‘risk preference in natural hazard insurance’ and ‘risk perception pre and post natural hazard’. The subject and text word searches were performed separately in all the databases and then combined with Boolean operators ‘OR’ and ‘AND’. Additionally, reference lists of relevant articles and related documents on databases of the UN and Intergovernmental Panel on Climate Change (IPCC) were also scanned for potentially relevant articles to ensure well-rounded search. To be eligible for inclusion, publications had to be in line with the inclusion criteria described in Table 1. Details of eligibility criteria, quality assessment and synthesis are summarised below.

**TABLE 1:** Inclusion and exclusion criteria.

Inclusion criteria	Exclusion criteria	Justification
Relevant to natural hazard insurance, risk perception, risk preference and decision-making under uncertainty	Not relevant to these	This is guided by the topic of the study
Behavioural economics, risk economics and psychology study field	Not in any of these fields	The cost mitigation strategies of insurance demand are a broad concept and limiting the study to these fields is to ensure the study focuses more on the behavioural side of the analysis
English language	Not in the English language	The authors of the study are only proficient in English language
Scientific work-peer reviewed	Not scientific work/no evidence of peer review	Because the study is mainly analysing scientific work that has been empirically tested and went through a rigorous review
Available in full text	Not available in full text	To ensure that the article includes its research protocol and steps
High impact factor publications preferred At least 10 citations	No impact factor with less than 10 citations	To ensure that the study captures the ongoing debate and relevant reviews on the topic in the literature by considering high impact papers

### Review principles: eligibility, inclusion and exclusion criteria

The study design had an inclusion and exclusion criteria as specified in Table 1. Types of study, in peer reviewed outlets, in English and with full articles available (accessible, were eligible for inclusion studies) were selected for inclusion from the papers identified by team members, using the inclusion criteria.

### Data extraction, synthesis and data analysis

#### Data extraction

The search and selection criteria included peer-reviewed publications which were identified using the search criteria terms as described above. During the search, 645 potential studies were identified, 100 were excluded based on their titles and abstracts. The remaining 545 papers were retrieved and reduced to 200 after removing duplicates and 39 papers were ultimately left after thorough assessment of the papers using the outlined inclusion and exclusion criteria.

**FIGURE 1:**
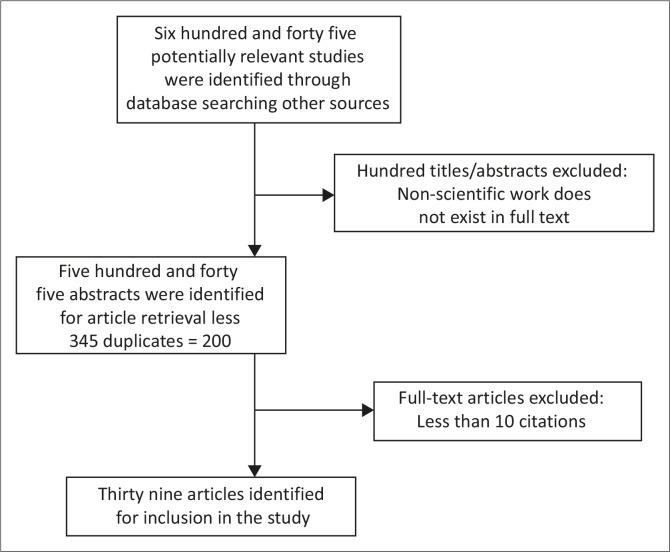
Preferred reporting items for systematic review and meta-analysis flow diagram for the retrieval of relevant studies.

This process is fully illustrated in Figure 1 below.

The screening procedure was guided by Higgins and Deeks’ framework (Colosia et al. 2016). All articles identified to be potentially eligible for inclusion in this review had full-text sourced. A data extraction form was used, which included the following elements: author(s), year of publication, title of study, country, study aim(s) or research question, study design, study setting (urban/rural), study population, sample size, key findings that relate to the review question, study limitations, implications and interpretations and conclusions from the authors. Data were extracted by the main author and accuracy checked by the second author. Studies with uncertainties about their inclusion were resolved by discussion.

#### Synthesis

These 39 papers were then reviewed and analysed using a narrative synthesis, with data synthesised and interpreted using sifting and sorting based on themes/key issues. A narrative synthesis approach as argued in the study by Howell et al. (2019) helps to summarise and identify patterns across studies using tabulations, textual descriptions, conceptual triangulation (concept mapping) and thematic analysis.

#### Data analysis

The extracted data were read and reread to identify common themes, methods and conclusions from the identified studies. Descriptive themes emerged and were discussed as part of the findings. The geographical precinct of the 39 studies is not restricted to a particular area, instead there are studies from USA, Australia, New Zealand, UK, Netherlands, Pakistan, Bangladesh, Norway, China, Germany, Venezuela, Austria, South Africa and Global studies.

### Ethical considerations

This article is a systematic literature review with no animals and humans involved, no primary data were collected; it is only a review of past studies. The article is written under the bigger project with ethics Number H20-BES-ECO-092 reviewed by the Research Ethics Committee (Human) at the University of Nelson Mandela, 29 June 2021.

## Results

Reviewing the studies and conflating with the study objectives, the following themes emerged for discussion around demand for natural hazard insurance: demand for natural hazard insurance; psychology of decision-making; risk perception; risk preference and willingness to pay. These are discussed in detail in the following subsection.

### Demand for natural hazard insurance

The demand for natural hazard insurance in developing economies such as Vietnam in Brouwer et al (2014); Reynaud and Nguyen (2018), Pakistan Abbas et al. (2015); Fahad and Jing (2018) and in china Liu et al. (2019); Tian, Yao and Jiang (2014); Wang et al. (2012) has identified that there is a low demand of natural hazard insurance. Reynaud and Nguyen (2018) identified that the demand for natural hazard insurance in developing economies has a low demand in the urban areas, whilst it is non-existent in the rural areas.

According to Reynaud and Nguyen (2018) in Vietnam, high demand is prevalent amongst big farmers and urban areas, and is low or negligible amongst subsistence farmers and rural areas. The slow penetration of insurance demand has been explained to have been caused by government aid, lack of awareness and adverse selection (Reynaud & Nguyen 2018). Roder et al. (2019) have noted that the mere existence of these natural hazard insurance has been affected by moral hazard. The studies of the demand for insurance of natural hazard can be classified based on the type of natural hazard from earthquake, flooding, drought and wildfires. A large number of literature in economics have analysed the demand for flood insurance such as in Vietnam, Navrud, Tuan and Tinh (2012) measure household willingness to contribute to flood prevention programmes. They find that households are willing to contribute on an average of 6.7 person-days per year and per household. Still, in Vietnam, Brouwer et al. (2014) investigate household willingness and ability to pay for flood micro-insurance.

They show that there exists a demand for flood insurance, even though a considerable share of the population indicates that they are unable to afford to pay for such insurance. Abbas et al. (2015) explore the household willingness to pay for flood insurance in flood-prone areas of Pakistan. Although a large proportion of household would be ready to buy flood insurance, their willingness to pay remains limited to around 0.27% of the mean monthly household income. More recently, Ren and Wang (2016) have estimated the willingness to buy flood insurance in rural China. They report that about two-thirds of the population would be ready to participate in a flood insurance programme. They also find that the influencing factors in the insurance demand include the recent frequency of floods, income and experience with lack of flood insurance.

### Psychology in natural hazard insurance uptake (psychology of decision-making)

When looking at the decision-making process of economic agents, Pasquini, Steynor and Waagsaether (2019) state that there are two types of decision-making approaches that economic agents undertake in the face of uncertainty which is a normative and descriptive approach. Pasquini et al. (2019) state that uncertainty can be defined by economic agents based on probability, delay in consequence or outcomes and the absence of information. According to Pasquini et al. (2019), the normative approach involves mathematical and analytical approaches in which individuals make rational decisions such as the Bayesian theory and expected utility theory. Whilst the descriptive approaches to decision-making try to look at the actual decision-making process such as the prospect theory and understand the flaws of the human mind, Pasquini et al. (2019) and Sum, Nordin and Akademia Baru (2018) analysed the decision-making biases in insurance purchasing and the study found that economic agents make heuristic decisions. According to Sum et al. (2018), heuristic decision-making process involves agents using shortcuts in making decisions in order to save time and because of limited cognitive capacities. The heuristic decision-making biases can be derived from four main issues which are (1) What you see is all there is, (2) Representativeness (3) Availability and (4) Effect (Pasquini et al. 2019; Sum et al. 2018).

Pasquini et al. (2019) state that availability is observed when economic agents make decisions based on the available memories and they measure the probability of likelihood based on those memories. Whilst Sum et al. (2018) state that representative heuristics is based on making decision of an event based on how much it represents a typical situation, for example, when tossing a coin with head and tail, agents are less likely to predict HHHHHHH than HTHTHTHT. Affect heuristics represents the bias based on affection and emotional feelings with what is good and what is bad experienced in relation to a stimulus (Sum et al. 2018). It is important to note that economic agents use three decision-making biases to analyse the uncertainty and their adoption strategy to mitigate effects. These biases include mental accounting versus how likely is the consideration of loss or gain (Sum et al. 2018). When looking at the psychology in natural hazard insurance, it is important to look at the salience of economic agents towards insurance as shown in the study by (Bordalo, Gennaioli & Shleifer 2012; Hu 2021; Västfjäll, Peters & Slovic 2014).

Hu (2021) states that the digitalisation of flood maps into the internet space and communication of economic agents on social media increased the salience of flood risks and increase in the uptake of flood insurance. Whilst Segal, Jong and Halberstadt (2018) found that the increase in the fusing effects of natural hazard in New Zealand as a function of fear encouraged prosocial behaviour and adaptation of costs mitigating programmes. It is important to note that Segal et al. (2018) found that the prosocial behaviour existed amongst the individuals who attributed the event to a super natural agency. According to Böhmelt (2020), the salience on natural hazard insurance increases just after an event and it attenuates after a short period after the event (Dumm et al. 2020) affirming the accession by stating that insurance take-ups spike just after the flood and then steadily decline to baseline. The study by Gallagher (2014) found that uptake from not affected areas also increased and is consistent with the Bayesian learning model that allows for forgetting or incomplete information about past events. Whilst Dumm et al. (2020) found that the effect attenuates as the losses fade from memory, the effect of losses on demand is much higher for more recent losses. According to Dumm et al. (2020), the representative heuristic model shows that individual policy holders overweight the probability of another catastrophic event occurring by nearly 50%, after such event has occurred.

### Risk (perception and preference)

Natural hazard insurance uptake and demand perception of the economic agents towards salience of natural hazard are analysed and the need to engage with adaptation methods to mitigate damages is important. Studies analyse the risk perception of economic agents related to climate and other natural hazards (Botzen, De Boer & Terpstra 2013; Carlton et al. 2016; Lujala, Lein & Rød 2015). Lujala et al. (2015) state that socio-demographic factors play a significant role in risk perception studies such as in the study by Habiba et al. (2012) who jointly analysed perception with socio-demographic factors such as gender, age and income level.

Whilst Lujala et al. (2015) looked at the affected area from a distance, they pointed out that in conjuncture with perception distance, it can be categorised into three sections which are spatial distance (physical)which affects perception, whilst temporal distance (refers to how soon people think of the effects of climate change) and social distance (refers to how people believe climate change affects people like them) have an impact on preference. According to Habiba et al. (2012), in his analysis to Bangladesh farming community, the study found that when looking at the mitigation programmes in terms of their adoption and absorption by the community, it is a two-step process.

Habiba et al. (2012) state that the two step process of the adoption and mitigation effectiveness involves accepting that the climate is changing and then adoption. Petrolia, Landry and Coble (2013) state that when looking at the demand for natural hazard insurance, the acceptance and understanding of the risk are important before the agents adopt which is insinuated in the (Habiba et al. 2012) two step adoption process. Reynaud, Aubert and Nguyen (2013) state that the coping appraisal is highly dependent on the threat appraisal level of fear in which it must reach a particular threshold for it to start. The coping appraisal includes several facets of perceptions which include the perceptions about one’s protective self-efficacy, response costs and action efficacy. According to Reynaud et al. (2013), the study adds the threat experience appraisal and the reliance on non-individual protection methods.

Carlton et al. (2016) looked at the USA and analysed the effects of extreme drought on climate change beliefs, risk perception and adoption attitudes and found that attitude and beliefs did not change, but the risk perception did change with agents being concerned about drought and pests more than flooding, whilst Lujala etal. (2015) found that gender educational level and political preferences do contribute towards an individual’s perception and attitude towards natural hazard issues. The results in the study by Lujala et al. (2015) also concur with the additional variable in PMT by Reynaud et al. (2013) because they found that personal experience of the events and their damage helps to change individual perspective and attitude, whilst Wachinger et al. (2013) tried to explain the reasons why there is a weak relationship amongst risk perception, attitude and personal action and found that there are three reasons which are experience and motivation, trust and responsibility and personal ability, in which personal ability includes economic and personal conditions.

Studies have looked at the changes in the risk preferences pre and post natural hazard events (Brouwer et al. 2014; Reynaud et al. 2013; Reynaud, Nguyen & Aubert 2018) using the Eckel and Grossman method to elicit risk preferences and used villages that did not experience even a control experiment and similar demographics and variables. Reynaud et al. (2013) found that there is heterogeneity in economic agents in terms of their preferences and they contribute significantly to understanding the demand of flood insurance, whilst a study by Cameron and Shah (2015) on earthquakes found that individuals who experienced the natural hazard are less likely to take risky options than individuals far away from the epicentre of the hazard. Cameron and Shah (2015) further state that the risk preferences vary with marital status with females being less likely to choose riskier options which Brouwer et al. (2014) concur with and state that choices and risk preferences are highly dependent on demographics.

### Willingness to pay

The studies of natural hazard insurance have been dissected into different types of natural hazards from cyclones, earthquakes Kellenberg and Mobarak (2011); Tian et al. (2014); Wang et al. (2012) to floods as shown in the study by Brody et al. (2017), Liu et al. (2019), Ren and Holly Wang (2016) and Reynaud et al. (2018). The demand for natural hazard insurance has been analysed by looking at the drivers for the willingness to pay such as risk preference, perspective and the price of the type of insurance. According to Reynaud et al. (2018), variable of the willingness to pay in the insurance sector is influenced by price and non-price factors such as the type of institution that is providing the insurance which mainly pertains to the trust issues of the consumers from government, non-profit making and private institutions, demographics and experiences of the events. The analysis of natural hazard insurance in China has been thoroughly considered in the literature Ren and Holly Wang (2016); Wang et al. (2012), Zhang and Qian (2018) with Zhang and Qian (2018) looking at the demand for earthquake insurance in the minority of the studies that identified the importance of demographics arguing that age is a significant variable in the determination of the willingness to pay. Zhang and Qian (2018) further state that experiences, higher risk perception, government aid and insurance experience have a high influence on the demand for earthquake insurance.

Whilst Tian et al. (2014) looked at the demand for earthquake insurance by focusing on the risk perception dimension, the study identified that the risk perceptions of earthquakes are influenced by the level of education and income level. Education and income have a significant impact on highly educated individuals who feel that they are less likely to be affected, whilst high income earners share the same sentiment with the idea that they have structures that are less likely to be affected by the hazard. Whilst the study on the demand for earthquake insurance in the USA looking at the willingness to pay, focuses on price factors, the results of the study found that the insurance demand is income inelastic and is perfectly price elastic (Arnal et al. 2016).

The literature also covers natural hazard insurance in the South Asian country of Pakistan Fahad and Jing (2018) and Abbas et al. (2015), focusing on floods and crop insurance, respectively. Fahad and Jing (2018) identified that in the rural areas, the demand for crop insurance is low to negligible, and that a uniform crop insurance is not ideal for the market, but rather fragments the market by crop type. Fahad and Jing (2018) further state that socio-economic demographics, nature of the hazard and the physical landscape of the area do influence the market significantly, whilst Abbas et al. (2015) looked at flooding insurance demand in the Pakistan market, analysing price, income and other non-monetary factors. The study by Abbas et al. (2015) found that flood insurance is income elastic and had a positive impact on willingness to pay for flood insurance. Off farm income has a positive impact on the consumer demand of flood insurance, and furthermore, this impact is also on monetary factors such as age of household head, land ownership and perception of the flood impact (Abbas et al. 2015). Tian et al. (2014) affirm the explanation by Fahad and Jing (2018) that the rural population dismissed the idea of the insurance policies, but they explain that the reason for this is the fact that their financial positions influence their decisions more.

Insurance and partial distance from the hazard are considered in studies by Botzen and Van den Bergh (2012) as well as Wang et al. (2012) and these studies identified that people who are staying in high risk and more vulnerable areas that are susceptible to flood have a low demand of flood insurance, and those in less vulnerable and protected areas have a high demand for flooding because of their understanding of the risk. These findings are contrary to the study by Liu et al. (2019) who found that the more susceptible one is to the hazard, the more the demand for insurance. A study of Germany and Netherlands on their demand for flood insurance found that the frequency of medium impact floods has a significant impact on decision-making (Seifert et al. 2013). Atreya, Ferreira and Kerjan (2015) argues that in the USA, the frequency is an important factor as it drives the purchasing of flood insurance, however this impact fades away after 3 years.

There are other studies that have tried to analyse the willingness to pay off these natural hazard insurances pre and post the event (Browne, Knoller & Richter 2015). The study for the Germany populace looked at the behavioural bias between the demand for bicycles and the demand for flood insurance (Browne et al. 2015). The analysis of this study is based on testing the hypothesis if risk exposure influences the demand for both types of coverage and understanding if there are any systematic differences between the two products (Browne et al. 2015). The individuals in Germany prefer high probability low consequence products over low probability high consequence products; however, these results might have been influenced by moral hazard which affects bicycle insurance market (Browne et al. 2015).

Whilst Raschky et al. (2013) looked at the level of uncertainty of government relief and how it can crowd out flood insurance, the results identified that the lower the uncertainty of the government relief, the more it crowds out flood insurance, which is a substitute product. Davlasheridze and Miao (2019) state that government assistance crowds out the flood insurance that has also been analysed in the USA and the results confirm the results by Raschky et al. (2013) and further note that these government reliefs affect the federal financial risk exposure to these natural hazards and climate change. These results are contrary to the results found in the study by Atreya et al. (2015) who test if mitigation efforts by the government and insurance uptake are substitutes.

Atreya et al. (2015) and Hu (2021) have looked at different perspectives of the willingness to buy and the demand for natural hazard insurance in the USA. Royal and Walls (2019) analysed the perception and understanding of the people in the USA Maryland of the risk of floods and also analysed if this influences their decisions of insurance choices. When looking at risk perspective, the theoretical underpinnings of studies depend on the Bayesian process of catastrophe experiences updating an individual’s perception and influencing their decision-making (Royal & Walls 2019). Royal and Walls (2019) found that the Bayesian process happens in Maryland, and individuals update their beliefs after every experience. The study further identified that individuals generally believe that they have a lower risk to flooding than their neighbour, which is contrary to the objective risk estimates which reflected that only 50% had a lower risk than other residents. Whilst Brody et al. (2017) tried to understand the motivation of coastal residence to voluntarily purchase federal flood insurance, they found the level of education, value of homes and the period of stay in the area as the major motivating factors to residence purchasing. Brouwer et al. (2014) further affirm the importance of education level insurance uptake. Interestingly, Royal and Walls (2019) found that the penetration of flood insurance is not highly influenced by the distance from the hazard areas.

In the USA, Hu (2021) found that salient behavioural factors influence insurance uptake and the study found that geographically distant peers are likely to purchase flood insurance if their peers have experienced floods. Alex (2019) looked at the likelihood of individuals having flood insurance, which increases with their social expectations and the results found that the increase in desire of social affirmation can be a driver for increase in the demand for flood insurance. In Australia, Han et al. (2020) state that sociological and communication studies identify that climate-related risks are specially produced and reproduced, and one cannot understate the importance of the salient factors.

## Discussion

The study undertook a systematic literature review of the attributes that derive the demand of natural hazard insurance looking at both the monetary and nonmonetary factors. Firstly, the study revealed how literature explained the psychological factors that influence the decision-making process of economic agents to buy natural hazard insurance. The study revealed that demand for insurance depends on the decision-making process which can be either normative or descriptive techniques. The study further shows that descriptive techniques which lead to heuristic decisions can be subjected to bias and experiences, which might in turn lead to the buying or the rejection of the insurance policy (Sum et al. 2018).

The study also expresses that the salience on the natural hazards sustains these policies over a long period and has a significant impact on demand of insurance. The study further shows that if the salience attenuates within a short period of time, this leads to a fall in the demand and also weakens the penetration of the natural hazard insurance with time (Hu 2021).

Literature pins protective motivation theory as the novel theory of changes in perspective and states that purchasing insurance is denoted as a coping appraisal. The results reveal that the risk perspective that leads to the purchasing of natural hazard insurance is dependent on the high level of education and income level; furthermore political affiliation has an impact on heuristic decisions of purchasing insurance. The study further identified that for the purchasing of natural hazard insurance as an adapting technique, one has to have a clear understanding of the threat and its level of uncertainty and the need to insure for it. Lujala et al. (2015) state that the increase in the belief and understanding of the natural hazards increases the demand for the flood insurance and increases the penetration rate of the natural hazards insurance products. Furthermore, demographic variables such as age have an impact on the perspective of decision-making which leads to purchasing insurance of natural hazards because of the frequency of experiences.

The results show that the understanding of the risk preferences of the economic agents is important to increase the demand for insurance. Reynaud et al. (2018) classify the economic agents as risk averse, and risk neutral and the risk a verse individuals are expected to purchase insurance to curb their risk exposure. According to Mahaprashasta, Mukhopadhyay and Pattanayak (2021), there are changes in risk preferences post a hazardous event and this will lead to increased purchases of natural hazard insurance, therefore there is increased post hazard penetration of insurance products. The results reveal that the literature explains the willingness to pay variable to be a comprehensive question that can be answered from a holistic perspective involving monetary factors such as price and income elasticity, domain covered (health, property, life, etc.), location and other sociodemographic factors (Botzen et al. 2013; Wagner 2019).

## Conclusion, identified gap for future studies

The study shows a holistic analysis of literature about natural hazard insurance demand and gives a comprehensive analysis of this literature and facets of the subject matter. The analysis also reveals the deficiencies in the literature, especially on the aspect of eliciting risk preference and empirical analysis of heuristic decision-making analysis. Furthermore, the study identified that the literature of natural hazard insurance has been widely found in developed economies and a few studies in developing economies and fewer studies in African countries and there is a need to further explore empirical studies on natural hazard insurance in African countries. The study also found that there is a need to explore several natural hazards and not to solemnly focus on flood insurance which has been widely explored in developing economies. The study is limited to a literature review and there is a need for regional or a country-specific analysis of the natural hazard insurance.
